# Physiological functions of SPP/SPPL intramembrane proteases

**DOI:** 10.1007/s00018-020-03470-6

**Published:** 2020-02-12

**Authors:** Torben Mentrup, Florencia Cabrera-Cabrera, Regina Fluhrer, Bernd Schröder

**Affiliations:** 1 Institute for Physiological Chemistry, Medizinisch-Theoretisches Zentrum MTZ, Technische Universität Dresden, Fiedlerstraße 42, 01307 Dresden, Germany; 2 Biochemistry and Molecular Biology, Faculty of Medicine, University of Augsburg, Universitätsstraße 2, 86135 Augsburg, Germany; 3 Biomedizinisches Centrum (BMC), Ludwig Maximilians University of Munich, Feodor-Lynen-Strasse 17, 81377 Munich, Germany; 4 DZNE–German Center for Neurodegenerative Diseases, Munich, Feodor-Lynen-Strasse 17, 81377 Munich, Germany

**Keywords:** Intramembrane proteolysis, Protein degradation, Signal peptide peptidase-like, γ-secretase, Signal transduction, Membrane trafficking

## Abstract

Intramembrane proteolysis describes the cleavage of substrate proteins within their hydrophobic transmembrane segments. Several families of intramembrane proteases have been identified including the aspartyl proteases Signal peptide peptidase (SPP) and its homologues, the SPP-like (SPPL) proteases SPPL2a, SPPL2b, SPPL2c and SPPL3. As presenilin homologues, they employ a similar catalytic mechanism as the well-studied γ-secretase. However, SPP/SPPL proteases cleave transmembrane proteins with a type II topology. The characterisation of SPP/SPPL-deficient mouse models has highlighted a still growing spectrum of biological functions and also promoted the substrate discovery of these proteases. In this review, we will summarise the current hypotheses how phenotypes of these mouse models are linked to the molecular function of the enzymes. At the cellular level, SPP/SPPL-mediated cleavage events rather provide specific regulatory switches than unspecific bulk proteolysis. By this means, a plethora of different cell biological pathways is influenced including signal transduction, membrane trafficking and protein glycosylation.

## Introduction

The concept of proteolysis was discovered more than 180 years ago, but it took around 160 years until it became evident that proteolysis can also occur within the plane of cellular membranes [1]. Proteases that catalyse substrate hydrolysis in cellular membranes are typically polytopic membrane proteins that arrange as pore-like structures allowing the concentration of sparse water in their catalytically active centres which are embedded in the hydrophobic membrane environment [2–4]. Most likely, lateral openings of the pore-like structure allow the substrates’ transmembrane (TM) domain to enter the core of the enzyme [5–7]. In addition to the limited water access, intramembrane proteases also face other difficulties: (1) substrates are embedded in lipids and thus access of the protease is much more difficult than in solution. (2) Diffusion rates in lipid environment differ from that in solution. (3) Lipid-embedded protein domains tend to form ordered helical structures further aggravating cleavage.

To date, four classes of enzymes that can cope with these challenges have been identified. Based on the amino acids or co-factors critical for catalysis, they are classified as metallo, serine, glutamyl and aspartyl intramembrane proteases [8]. While in mammals, so far, S2P [9] and Rce1 [10] represent the only members of the metallo and glutamyl intramembrane classes, respectively, various members of the Rhomboid family [11] constitute the class of serine intramembrane proteases. The aspartyl intramembrane proteases are constituted by two protease families: the presenilins [12] and the Signal-Peptide-Peptidase (SPP)/SPP-like (SPPL) family [13]. The two presenilins constitute the catalytic subunit of the high molecular weight γ-secretase complex that is involved in the processing of Notch and the β-Amyloid-Precursor Protein (APP). Thus, the presenilin family is critically involved not only in development but also in the pathology of Alzheimer disease.

However, more recently it became evident that members of the SPP/SPPL family are also important players at the interface of membrane protein homeostasis and signal transduction and execute important functions in the living organism [8]. This is reinforced by a strong conservation through all kingdoms of life [14]. In mammals, the SPP/SPPL family is constituted by five members. SPP is the first discovered member of the family and encompasses a KKXX-motive at its C-terminus, which is most likely involved in retaining the protein in the Endoplasmic Reticulum (ER) [15]. SPPL3 is the smallest member of the family and resides in the Golgi apparatus [16]. The three SPPL2 proteases complete the SPP/SPPL family: SPPL2a localises to endo-/lysosomal compartments where it is directed to by a canonical tyrosine-based sorting motif in its C-terminus [17]. SPPL2b is mainly detected at the plasma membrane [16, 17], while SPPL2c resides in the ER and ER-Golgi intermediate compartment (ERGIC) [16, 18]. So far, however, what kind of signal retains SPPL2c in these early secretory compartments remains enigmatic.

Unlike the members of the presenilin family, which require the formation of high molecular weight protein complexes to gain proteolytic activity, all members of the SPP/SPPL family seem to be catalytically active without additional co-factors. This finding is based on overexpression studies, which allow an increase in SPP/SPPL activity by sole expression of the protease, whereas increased presenilin activity requires co-expression of all complex components [14, 19]. However, there are indications that some members of the SPP/SPPL family undergo multimerisation or association with other cellular proteins that are present in the cell in excess amounts. SPP was observed as homo-dimer [20, 21] or tetramer [22]. Furthermore, SPP forms complexes with other cellular proteins, for instance Derlin 1 and the E3 ubiquitin ligase TRC8, which play a role during ER-associated degradation (ERAD) and allow recognition and cleavage of substrates that would otherwise not be processed by SPP, such as the unfolded protein response regulator XBP1u [23]. Consequently, SPP has been observed in distinct high molecular weight protein complexes when analysed by native gel electrophoresis [18, 24]. Interestingly, the other ER-localised SPP/SPPL protease, SPPL2c, is also part of a large, approximately 500 kDa protein complex. However, at least in murine testis the observed molecular weights of SPP and SPPL2c-containing complexes differ significantly [18]. So far, the composition as well as the function of these high molecular weight assemblies remain unknown. The ability of other family members to undergo similar protein interactions has not been investigated.

## Cleavage mechanism of SPP/SPPL intramembrane proteases

The architecture of the catalytic centre is predicted to be conserved among all SPP/SPPL family members as well as in the presenilins [14]. The two aspartyl residues critical for hydrolysis are located in TM domains 6 and 7 of all SPP/SPPL proteases and are embedded in the highly conserved signature motives (Y/F)**D** and G(L/I/F)G**D** [25, 26]. Mutation of either aspartyl residue abolishes the catalytic activity of all SPP/SPPL family members [16, 18, 27–31]. An equally conserved Q**PAL**LY motif in TM domain 9 completes the catalytic site architecture. Mutations in this so-called PAL-motif negatively affect the catalytic activity of SPP as it is observed for the presenilins [32], where it was found to bind to a β-strand within APP and may therefore be involved in substrate recognition [7]. Formal proof that the PAL-motif is also critical for the other SPPL family members, however, is so far pending.

Co-crystallisation of γ-secretase, the catalytically active complex of presenilin, with its substrates Notch and APP, indicates that aspartyl intramembrane proteases have evolved a β-strand close to the catalytic aspartyl residues that interacts with β-strands in the substrate TM domain, allowing access to the substrates’ peptide-bonds [6, 7]. Since neither the β-strand close to the protease active site nor the β-strands in the substrates have been observed when structures of protease and substrate were analysed separately [3, 33], it may be speculated that β-strand formation in both protease and substrate are induced upon binding. By this mechanism aspartyl intramembrane proteases may improve access of the catalytic centre to the peptide bond that is hydrolysed, thus coping with the challenge that the TM domains of their substrates tend to form ordered helical structures within cellular membranes. Given the similarities of the catalytic centres between presenilins and the SPP/SPPL family as well as predictions of secondary structures it is likely that SPP/SPPL also utilise inducible β-strands to gain substrate accessibility, however experimental proof for this is still missing.

Interestingly, presenilins and also rhomboid proteases were shown to be extremely slow enzymes with, for instance, a *k*_cat_ of 6.0 h^−1^ for proteolysis of APP by presenilin in cultured cells [34, 35]. An explanation for this remarkably slow catalysis may be substrate recognition that needs multiple structural requirements to be fulfilled. Since not only diffusion rates within a lipid bilayer differ from that in solution but also protein dynamics, it is well imaginable that it takes time until the correct conformation of a substrate in the catalytic centre is achieved.

Similar to presenilins, SPPL2b seems to apply a multistep processing to finally release its substrate from the membrane as indicated by data on cleavage of TNFα [28, 36]. Starting with an initial cleavage that releases a C-terminal substrate peptide to the extracellular space, SPPL2b proceeds with multiple consecutive cleavages in N-terminal direction to release an intracellular peptide (ICD) to the cytosol [36]. The latter is also referred to as processivity of the enzyme. However, it is not clear yet, whether initial cleavage and processivity of SPPL2b depend on different determinants within a substrate. Although a variety of initial cleavage sites within SPPL2b and also SPPL2a substrates have been determined [28, 37–40], no obvious consensus sequences were identified. In contrast, analysis of cleavage sites in SPPL3 substrates indicates that methionine and tyrosine in the P1 position are favoured [41], but it still remains to be proven by mutational analysis whether a true consensus sequence for SPPL3 exists or if rather secondary structure elements determine cleavability. Data on SPP suggest that it may apply a similar cleavage strategy as SPPL2b [42]. Whether the remaining members of the SPP/SPPL family also recognise and process their substrates similarly and how the lipid environment impacts on substrate processing by SPP/SPPL proteases, remains to be investigated.

## Substrate spectrum of SPP/SPPL proteases

**Fig. 1:**
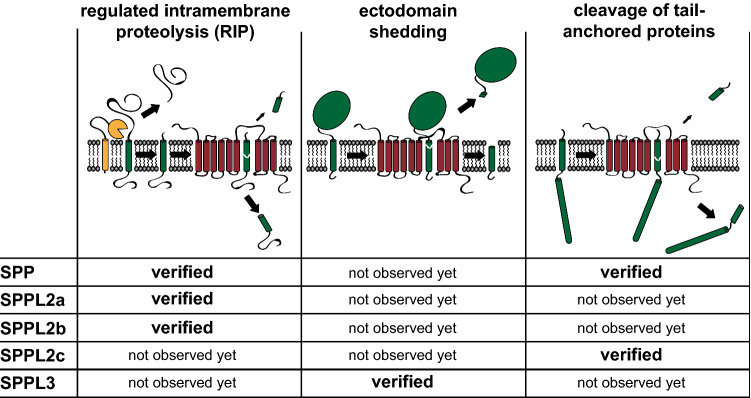
Major cleavage modes of SPP/SPPL proteases. Summary of the current state of knowledge of accepted substrate types and the major different modes of substrate cleavage by signal peptide peptidase (SPP) and the four SPP-like proteases SPPL2a, SPPL2b, SPPL2c and SPPL3

The biological function of a protease is largely defined by its substrates. Presenilins/γ-secretase have been shown to cleave more than hundred substrates [43] and exhibit a selectivity for type I membrane proteins [8]. Despite many similarities, presenilins and SPP/SPPL proteases exhibit opposing orientations within the membrane. While the N-terminus of presenilin resides in the cytosol, the N-terminus of SPP/SPPL proteases faces the lumen of cellular organelles or the extracellular space at the plasma membrane [15]. This leads to an opposite orientation of the catalytic centre including the potential β-strand in SPP/SPPL proteases as compared to presenilins and may explain why SPP/SPPL family members only accept substrates with type II-oriented transmembrane segments with the N-terminus facing the cytosol. In comparison to presenilins/γ-secretase [43], the list of SPP/SPPL substrates is still rather short—though continuously growing. A comprehensive substrate list was part of a recent review [8]. However, so far not all substrates identified in cellular substrate-protease co-expression assays have been confirmed under endogenous conditions in vivo, e.g. using SPP/SPPL-deficient mice. This is in part due to the lack of available mouse models, e.g. for SPP as well as the lack of specific antibodies against the N-termini of the proteins. It should be emphasised that such cellular assays rather determine general cleavability but fail to proof functional relevance. Beyond the universal requirement of a transmembrane segment in type II orientation, the layout of the substrates differs with regard to the size of the extracellular/luminal domain. Individual SPP/SPPL family members vary in their ability to accept substrates with larger ectodomains and therefore also in their capacity to perform different cleavage modes (Fig. 1), which will be introduced in the following. Beyond these three discussed scenarios, further types of processing may occur. The yeast orthologue of SPP, for instance, mediates degradation of the zinc transporter Zrt1, a polytopic membrane protein, which seems to indicate a direct ability of this protease to cleave substrates with multiple transmembrane segments [44]. However, this has not been documented yet for mammalian SPP/SPPL proteases.

### Regulated intramembrane proteolysis of type II membrane proteins

This cleavage mode is exemplified by Tumor Necrosis Factor α (TNFα), one of the first SPPL substrates identified [16, 28]. TNFα is a type II transmembrane protein that is transported to the cell surface, where the bulk of its ectodomain is removed by TNFα-converting enzyme ADAM17 and other proteases commonly termed sheddases [45]. This proteolytic processing step also known as ectodomain shedding results in the release of the TNFα ectodomain, which acts as a central proinflammatory cytokine in the body [46]. At the same time, a short N-terminal TNFα fragment (TNFα NTF) remains in the plasma membrane of the TNFα expressing cell. In a second proteolytic step, this TNFα NTF is hydrolysed by SPPL2a or SPPL2b within its TMD domain, resulting in the release of an intracellular fragment, the TNFα ICD, and a secreted peptide, the TNFα C-Peptide [16, 28]. This two-step proteolytic processing is also known as Regulated Intramembrane Proteolysis (RIP), since the first cleavage step is required for the second step to occur. Furthermore, in this model regulation is considered to occur primarily at the first proteolytic event. Subsequently, the constitutively active intramembrane proteases continue to process the remaining membrane-bound fragments. Beyond ectodomain shedding, also intracellular proteolytic pathways can generate NTFs from type II transmembrane segments. In case of CD74 [47], luminal lysosomal proteases shorten and degrade the substrate’s ectodomain rather than producing a defined secreted cleavage fragment. Importantly, also the first discovered function of SPP, the processing of signal peptides [27], follows the RIP cleavage mode. The release of the signal peptide from the nascent protein by signal peptidase is considered to be a prerequisite for further processing by SPP. Presenilins, SPP, SPPL2a and SPPL2b seem to prefer substrates with a rather short ectodomain (max. 60 amino acids). Thus, the substrates so far characterised either undergo Regulated Intramembrane Proteolysis or occur with naturally short ectodomains [45]. However, this does not rule out that these proteases are able to cleave proteins with longer ectodomains under certain conditions or with regard to specific substrates. This was shown for SPP, which in complex with the rhomboid pseudoprotease Derlin-1 and the E3 ubiquitin ligase TRC8 is able to cleave the unfolded protein response regulator Xbp1u [23]. However, reports that similar mechanisms are utilised by SPPL2a, SPPL2b or SPPL2c are currently pending.

### Ectodomain shedding by SPPL3

In contrast to the other members of the SPP/SPPL family, SPPL3 has no preference or requirement for a short substrate ectodomain [30, 41, 48]. It efficiently cleaves a wide spectrum of type II membrane proteins independent of the length of their C-terminal domains and, consequently, does not depend on a regulatory preceding cleavage. With this property, SPPL3 so far stands out from the family of aspartyl intramembrane proteases, and behaves more like rhomboid proteases [49]. Moreover, since the regulatory step of proteolysis is missing, the question about how SPPL3 activity is regulated arises. So far, it is believed that intramembrane proteases, once matured, are constitutively active, which would suggest, that regulation of this proteases must occur on its expression level or through altered degradation. But, based on the finding that SPP can associate with other proteins to change its substrate specificity [23], interaction with certain unknown co-factors must also be considered as a regulatory mechanism. Importantly, the consequence of SPPL3-mediated cleavage is the secretion of the released substrate ectodomains. Therefore, the biological function of SPPL3 resembles that of classical sheddases like members of the A Disintegrin and Metalloprotease (ADAM) family.

### Cleavage of tail-anchored proteins

As a third cleavage mode, recent discoveries have added tail-anchored (TA) proteins, also referred to as type IV membrane proteins so the list of SPP/SPPL substrates [8, 50]. In principle, TA proteins reflect proteins with a transmembrane segment in type II orientation and a very short luminal/extracellular domain usually comprising just a few residues and thus perfectly fulfil the criteria to become SPP/SPPL substrates. A variety of ER-localised TA proteins have been identified as SPP substrates, among them Heme oxygenase 1 (HO-1) [50, 51]. Most recently, also SPPL2c, up to then an orphan protease, was shown to cleave certain TA proteins in the ER [18, 31, 52]. Whether other members of the SPP/SPPL family also accept TA proteins as substrates remains subject for further studies.

## Substrate selection by SPP/SPPL proteases

When SPP/SPPL proteases were identified in 2002, it was hypothesised that they more or less non-specifically cleave type II membrane proteins, acting as a kind of membrane proteasome that releases proteins and their fragments from the membrane to ensure homeostasis [53]. Meanwhile, we know that SPP/SPPL proteases are more selective than initially thought and that a type II membrane topology is a necessary but not sufficient requirement for substrate cleavage [54]. This raises the question how SPP/SPPL proteases select their substrates. For SPPL2a and SPPL2b it has been shown that other than the length of the ectodomain, most likely structural, determinants within the two juxtamembrane (JM) domains as well as helix destabilizing residues with in the TM domain of the substrate determine its cleavability [40, 54, 55]. However, in most cases mutagenesis of single or selected groups of residues failed to turn a *bona fide* SPP/SPPL substrate into a non-substrate, suggesting that multiple components define the quality of a substrate.

Also for cleavage of TA proteins by the two ER-resident proteases SPP or SPPL2c, the TA topology per se is not sufficient [18, 31, 50], since only selected TA proteins are processed. Most intriguingly, the spectrum of TA proteins cleaved by either SPP or SPPL2c in co-expression assays was overlapping only partially. SPPL2c is capable of cleaving the SPP substrates HO-1 and RAMP4-2 [18]. However, it fails to efficiently proteolyse the closely related RAMP4 protein, which is a substrate of SPP [18]. Furthermore, SPPL2c did also not process the Hepatitis C virus (HCV) core protein or Xbp1u. This strongly indicates that recognition of substrates occurs in a protease-specific way and that SPP and SPPL2c, despite sharing the same intracellular localisation, fulfil unique biological functions. Based on the so far identified substrates, SPPL2c, similar to SPP, SPPL2a and SPPL2b, seems to have a preference or requirement for substrates with short ectodomains. Intramembrane proteolysis may not only be affected by complex formation of the protease, but also by that of substrates. HO-1 though being catalytically active in a monomeric state was found to form dimers and oligomers in the ER. A W270N mutation within the transmembrane segment reduced HO-1 oligomerisation, but at the same time increased intramembrane proteolysis [56]. This suggests that only the monomer of HO-1 is cleaved and that oligomerisation of HO-1 may prevent access of SPP. To better define the molecular mechanisms that allow SPP/SPPL proteases to discriminate substrates from non-substrates is an important question for future work. But perhaps even more critical is to understand how the cleavage of individual substrates is regulated. Since the cleavage by SPPL3 or the processing of TA proteins by SPP and SPPL2c occur directly without any preceding shedding, alternative regulatory mechanisms acting directly on the intramembrane proteases have to be assumed.

## Cell biological functions of SPP/SPPL-mediated proteolysis

In general, proteolysis can fulfil degradative functions for substrate proteins. However, in other cases, cleavage occurs as a specific posttranslational modification selectively altering the properties of the substrate protein. The selectivity of SPP/SPPL-mediated proteolysis argues against these proteases being a universal proteostatic mechanism for type II or TA proteins. Nevertheless, for their substrates SPP/SPPL proteases do fulfil a proteostatic function demonstrated by an accumulation of the non-cleaved substrates [18, 31, 39, 52, 57] which can reach major amounts like that of the CD74 NTF in SPPL2a-deficient B cells [57]. This substrate accumulation indicates that alternative proteostatic pathways cannot immediately take over degradation of these proteins. In case of TA proteins or SPPL3-cleaved type II membrane proteins, the abundance of the full-length substrate proteins increases in the absence of the proteases [18, 31, 48]. In contrast, NTFs from those substrates undergoing RIP accumulate if the intramembrane cleavage is blocked [39, 57]. All accumulating proteins or protein fragments are integral to the membrane. Functional consequences of this protein accumulation depend on the nature and molecular function of the specific substrate protein.

**Fig. 2:**
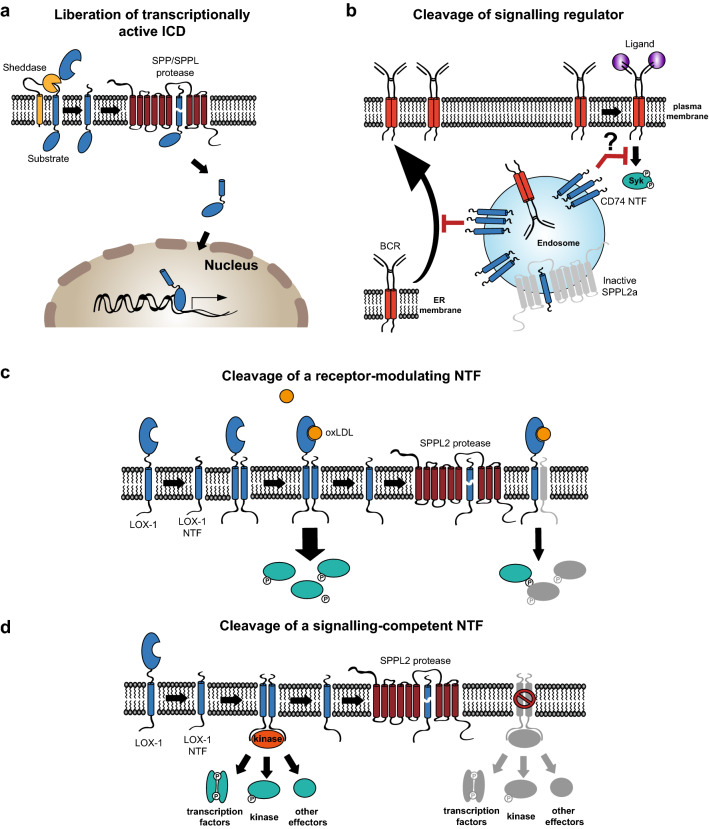
Mechanisms of signal transduction regulation by SPP/SPPL proteases. **a** SPP/SPPL proteases can transduce intracellular signals by releasing an intracellular domain (ICD) which either acts as transcription factor itself or is able to exert an impact on gene expression by interaction with the transcriptional machinery. **b** As exemplified by processing of CD74, SPP/SPPL proteases can cleave regulatory components of signalling pathways. The N-terminal fragment (NTF) of CD74 (depicted in blue) has the capability to influence subcellular trafficking of the B cell receptor (BCR, shown in red) and presumably also its downstream signalling. Since SPPL2a is required for clearance of the CD74 NTF, activity of this protease indirectly has a major impact on signal transduction in B cells. **c**, **d** SPP/SPPL proteases can also directly be involved in proteolytic processing of active receptor proteins like the Lectin-like oxidised low-density lipoprotein receptor 1 (LOX-1). In this case, the receptor NTFs can act as enhancers of the signaling of the full length receptor, which is induced by oxidised LDL (oxLDL) (**c**). Thus, turnover of the NTF controls LOX-1 signalling. Furthermore, the LOX-1 NTF was found to be an active signalling protein itself (**d**). In an autonomous way, most likely without the need of a ligand, this fragment activates MAP kinases. Therefore, the cellular levels of this fragment—controlled by SPP/SPPL proteases—are a direct determinant of the resulting signalling activation

In addition to substrate accumulation in the membrane, loss or inhibition of SPP/SPPL-proteases abolishes generation of the cleavage fragment. However, in many cases the fate and even more the function of the respective cleavage fragments remain enigmatic. For those proteins undergoing RIP, in particular the function of the ICDs released into the cytosol has been analysed in analogy to the Notch pathway where the ICD acts as a transcription factor [58]. Whereas the Notch ICD has a size of ~ 85 kDa, the ICDs of the so far identified SPP/SPPL RIP substrates with the exception of Teneurin-1/ODZ1 [59] comprise less than 100 amino acids. Presumably due to their small size in combination with rapid turnover, they are intrinsically difficult to detect by biochemical means. Using a cell-based reporter assay, the general ability of the ICDs from CD74, TNFα and ITM2b [60] to enter the nucleus was confirmed. An impact on transcriptional regulation has so far only been reported for the ICDs of TNFα [16] and CD74 [60, 61]. The ICD of the latter has been linked to the signal transduction of the cytokine Macrophage migration inhibitory factor (MIF) which binds to CD74 [47]. The release of a transcriptionally active ICD is probably the most direct way by which intramembrane proteases can be involved in cellular signal transduction (Fig. 2a). Cleavage of TA proteins by SPP or SPPL2c does not always lead to a well-detectable size difference of precursor and cleavage fragment due to the close proximity of the cleavage site to the C-terminus of the protein, which complicates their discrimination by Western blot analysis. However, the protease expression leads to an overall reduction of the substrate in the cell [18, 31, 50, 52] which indicates that the half-life of the cytosolic cleavage product is significantly shorter than that of the membrane-bound precursor. Therefore, the intramembrane cleavage may be regarded as the initiating step for the turnover of these TA proteins [62]. However, at least in the case of HO-1, the cleavage fragment exhibits certain stability after its release from the membrane and can be detected in the cytosol as well as in the nucleus [18, 51, 63]. For the other TA proteins cleaved by SPP or SPPL2c a role of the cytosolic cleavage fragments has not been investigated yet.

Beyond the ICDs, even less is known about cleavage fragments released to the luminal or extracellular side of the membrane. In case of TA proteins these will comprise only a few residues and NTFs from most RIP substrates usually exhibit luminal domains of less than 50 amino acids. In contrast to the poorly characterised cleavage fragments, work over the last years has put together a picture that a major if not the primary purpose of SPP/SPPL-mediated proteolysis is to control the levels of the membrane-bound substrate proteins in the cell. By this means, the activity of these proteases has a major regulatory impact on central cellular pathways, which are reviewed in the following.

### Regulation of signal transduction

SPP/SPPL proteases also influence signal transduction directly at the membrane. This is exemplified by CD74, the invariant chain of the MHCII complex [47]. In vivo, SPPL2a has a leading role in the cleavage of CD74 NTFs [64] demonstrated by its massive accumulation in B cells and dendritic cells from SPPL2a-deficient mice [57, 65, 66] and humans [67, 68]. At the cellular level, the functional consequences of the accumulating CD74 NTF have so far only been analysed in murine SPPL2a-deficient B cells [69]. In these cells, the signalling of the B cell antigen receptor (BCR) which is not only important to activate B cells in an antigen-specific manner, but also provides essential survival signals during differentiation, is compromised in a CD74 NTF-dependent manner. Interestingly, in particular the BCR-mediated activation of the PI3K/Akt pathway is impaired when SPPL2a is non-functional, which is reflected in altered inactivation of Foxo transcription factors. So far, no direct signalling-activating capacity of CD74 has been reported. In its role as MIF binding receptor it depends on co-receptors like CD44 to initiate intracellular effects [47]. In this regard, the CD74 NTF may be considered as an indirect regulator of BCR signalling (Fig. 2b). In part, the impaired BCR signalling in *SPPL2a*^*−/−*^ B cells may be explained by a CD74 NTF-induced mis-trafficking of the receptor. However, since BCR-triggered MAPK activation was unaffected in these cells [69] also additional mechanisms more directly interfering with the PI3K/Akt pathway seem possible.

In addition to this rather indirect impact on signal transduction, SPP/SPPL proteases can also process active signalling components like the Lectin-like oxidised low-density lipoprotein receptor 1 (LOX-1). LOX-1 is one of the major receptors for oxidised LDL (oxLDL) in the vasculature [70]. Beyond mediating oxLDL uptake into vascular cells, it triggers various signalling pathways including MAP kinases and NFκB and thereby activates endothelial cells, which can initiate endothelial dysfunction and atherosclerotic plaque formation [70]. Different proteolytic pathways including ectodomain shedding by ADAM10 and cleavage in lysosomes generate membrane-bound LOX-1 NTFs, which are then further processed by SPPL2a/b [39]. In contrast to CD74 processing, both SPPL2a and SPPL2b contribute to LOX-1 proteolysis under endogenous conditions. The generation of the NTF was found to be independent of LOX-1 ligands and primarily reflects constitutive turnover of the receptor. Importantly, this receptor fragment significantly accumulated in aortae from SPPL2a/b double-deficient mice [39]. The LOX-1 NTF, which is controlled by SPPL2a/b, can influence pro-atherogenic signalling pathways by two different mechanisms (Fig. 2c, d). First, the LOX-1 NTF interacts with the full-length LOX-1 protein and by this means enhances oxLDL-induced signalling of this receptor (Fig. 2c). Second, the LOX-1 NTF is capable to induce autonomous signalling independent of the full-length LOX-1 and oxLDL (Fig. 2d). The latter appeared to be linked to its ability to associate and oligomerise with other NTF molecules via the transmembrane segment. This NTF-induced signalling primarily activated ERK and p38 MAP kinases, but not the NFκB or PI3K/Akt pathway. Further downstream, this resulted in the activation of a pro-atherogenic and pro-fibrotic transcriptional program. As this study has revealed, cellular levels of the LOX-1 NTF are controlled by the intramembrane proteases SPPL2a/b [39]. Since the LOX-1 NTF fulfils a dual function as signalling enhancer of oxLDL-induced signalling as well as autonomous activator of MAP kinases, SPPL2a and SPPL2b negatively regulate both of these signalling modes. This puts these proteases in direct control of a central axis promoting endothelial activation and later dysfunction in the context of atherosclerotic plaque development with important pathophysiological implications.

**Fig. 3:**
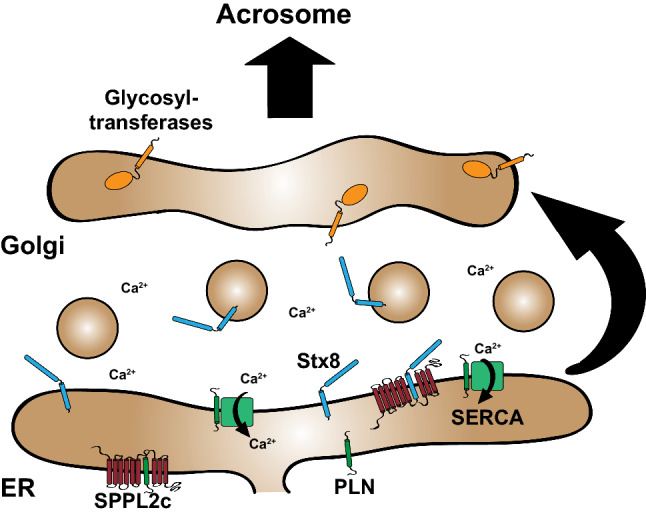
The role of SPPL2c in the murine testis. SPPL2c shows selective expression in elongated spermatids. In these cells, SPPL2c resides in the endoplasmic reticulum (ER). Two in vivo substrates of this protease have been identified: the SNARE protein syntaxin 8 (Stx8) and Phospholamban (PLN), an interactor and inhibitor of the SERCA Ca^2+^ ATPase. Both proteins accumulate in the testis of SPPL2c-deficient mice. As functional consequences of SPPL2c-deficiency, handling of Ca^2+^ and membrane trafficking in the secretory pathway are altered in spermatids. *SPPL2c*^*−/−*^ spermatids show a reduced cytoplasmic Ca^2+^ concentration. Furthermore, the organisation of the Golgi apparatus is less compact which may have implications for the protein delivery to the forming acrosome. Mature *SPPL2c*^*−/−*^ sperm cells show an altered glycosylation pattern presumably reflecting alterations of glycosyltransferase trafficking which has been characterised in SPPL2c-overexpressing HEK cells

SPPL3 has been linked to calcium-dependent signalling. Ca^2+^ represents an important second messenger, which can be released from intracellular stores in the ER [71]. In a screen SPPL3 was identified as an activator of the transcription factor NFAT [72]. SPPL3 enhances the store-operated calcium entry (SOCE) via the plasma membrane. When the Ca^2+^ stores in the ER become depleted upon cellular activation, the protein STIM1 (stromal interaction molecule 1) activates the Orai calcium channel in the plasma membrane. SPPL3 binds to STIM1 and thereby enhances its interaction with and activation of Orai. Interestingly, this function of SPPL3 is independent of its catalytic activity [72]. In the Jurkat T cell line, knockdown of SPPL3 impaired T cell receptor induced Ca^2+^ influx and NFAT activation. If and to what extent activation of T cells and T cell-dependent immune responses are affected in *SPPL*3^*−/−*^ mice has not been reported yet. Interestingly, also SPPL2c has been implicated in regulation of cellular Ca^2+^ (Fig. 3). Filling of the ER Ca^2+^ stores and also clearance of Ca^2+^ from the cytoplasm depends on the ER-resident Ca^2+^-ATPase SERCA1/2, which can be regulated by the small ER-resident TA protein phospholamban as it has been well characterised in the heart, but also skeletal muscle tissue [73, 74]. PLN was recently identified to be a substrate of SPPL2c and accumulates in SPPL2c-deficient spermatids, a specific stage of differentiating male germ cells, where SPPL2c is expressed [18]. These cells showed reduced cytosolic Ca^2+^ concentrations, which demonstrates an alteration of Ca^2+^ handling. However, how this is precisely linked to the PLN accumulation as well as the down-stream effects remain to be investigated.

### Regulation of membrane trafficking

SPP/SPPL proteases can directly influence membrane trafficking by cleaving N-ethylmaleimide-sensitive factor attachment protein receptor (SNARE) proteins which facilitate membrane fusion [75]. Proteomic screens identified different SNARE proteins as substrates of SPPL2c and SPP [18, 31, 52]. Upon overexpression in HEK cells, SPPL2c cleaves and thereby depletes the SNARE or SNARE-interacting proteins VAPA, VAPB, VAMP8, syntaxin (Stx) 5, Stx8, Stx18 and Membrin, which reside in the ER and other parts of the early secretory pathway [31]. In a similar set-up, SPP processes Stx18, but not Stx5, which is followed by proteasomal degradation of the resulting cytosolic cleavage fragment [52]. This suggests only a partial overlap of SNAREs proteins cleaved by SPPL2c and SPP, although several of the mentioned SPPL2c substrates may not have been tested for cleavage by SPP yet. In agreement, also the effects of SPP and SPPL2c overexpression on membrane trafficking are distinct [31, 52]. In general, it can be assumed that proteolysis of ER- or Golgi-localized SNARE proteins abolishes their function in membrane fusion. In SPPL2c-overexpressing cells, transport of cargo from the ER to the Golgi was impaired leading to its accumulation in the ER so that secretory and membrane proteins remained immature [31]. This also prevents glycosyltransferases from reaching the Golgi apparatus where they reside. Consequently, the synthesis and trimming of glycan side chains of glycoproteins is significantly altered when SPPL2c is expressed. Over time, SPPL2c expression in cultured cells can induce the disassembly of the *cis*- and medial Golgi [31]. Since SPPL2c expression can have such a critical impact on the secretory pathway it may explain why SPPL2c is not found to be expressed in normal somatic cells, but only in differentiating male germ cells, where it helps to reorganize the cellular compartments during sperm maturation as discussed below (Fig. 3). Stx8 accumulates in the testis of SPPL2c-deficient mice, which confirms that SPPL2c-mediated SNARE cleavage occurs in vivo. Whether other substrates identified in protease-overexpressing cells play a role in this context remains to be characterized.

In contrast to SPPL2c which may change Golgi morphology, in cells overexpressing SPP especially the ER is affected. SPP, but not its catalytically-inactive mutant, induces a re-organization of the ER characterized by the formation of densely packed, but highly dynamic ER clusters [52]. This is associated with a suppression of ER-microtubule interactions. The access of cytosolic proteins and also of translating ribosomes to the ER cisternae in the centres of the clusters is impaired. Nevertheless, the functionality of the secretory pathway appears to be largely preserved [52]. Despite these striking effects of SPP overexpression on ER morphology, it is unclear yet, to what extent endogenous SPP activity is required to maintain or regulate organisation of the ER. Furthermore, it is unknown if the cleavage of Stx18 by the ubiquitously expressed SPP [15] is a constitutive process or occurs in a regulatory manner to re-arrange the ER.

The impact of SPPL2a-deficiency on membrane trafficking in murine B cells is rather indirect in comparison to the direct cleavage of SNARE proteins by SPP and SPPL2c. *SPPL2a*^*−/−*^ B cells show an accumulation of endosome-derived vacuoles which is completely rescued in *SPPL2a*^*−/−*^* CD74*^*−/−*^ mice [57]. This clearly identifies the non-cleaved CD74 NTF as the causative element. A capability of CD74 to impair trafficking and to induce endosomal enlargement has been observed previously [47]. Furthermore, cathepsin S-deficient B cells, where cleavage of the CD74 luminal domain is impaired and slightly longer CD74 NTFs accumulate, exhibit a vacuolation-phenotype similar to *SPPL2a*^*−/−*^ B cells, however, less pronounced [76]. The obvious morphological changes in the endocytic system of *SPPL2a*^*−/−*^ B cells also have functional implications [69, 76]. The degradation of endocytosed fluid phase cargo is delayed. At the same time, the internalisation rate of the BCR is enhanced, which is reflected in a partial shift of the receptor pool from the plasma membrane to intracellular compartments. This altered BCR trafficking and the resulting reduced surface abundance may represent a major mechanism of the impaired BCR signalling [69]. Mistrafficking of receptors in *SPPL2a*^*−/−*^ B cells is not limited to the BCR since also the receptor for the survival-promoting cytokine BAFF exhibits reduced levels at the plasma membrane [57]. All so far observed changes in the endocytic system of *SPPL2a*^*−/−*^ B cells are strictly CD74-dependent raising the question regarding the underlying mechanism.

### Regulation of protein glycosylation

A distinctive feature of many secreted and membrane proteins, but also of resident proteins of the endo-/lysosomal system is glycosylation. On their way through the secretory pathway various sugar moieties are added either on asparagine residues, termed *N*-glycosylation, or on serine/threonine residues, termed *O*-glycosylation. In eukaryotic organisms, *N*-glycan synthesis is initiated in the ER resulting in high-mannose type glycans attached to the luminal domain of secretory and membrane proteins [77]. Within the Golgi apparatus numerous glycosyltransferase and glycosidases compete for these high-mannose type precursor glycans converting them into higher-order, complex *N*-glycans [78, 79]. These glycosyltransferases are type II membrane proteins with a short N-terminus, while the glycosyltransferase activity is located in the rather large C-terminal ectodomain [78, 80]. SPPL3 accepts a variety of these glycan modifying enzymes as substrates and releases their catalytic domain from the Golgi membrane, which leads to secretion of soluble glycosyltransferases [41, 48]. Although these soluble glycosyltransferases released by SPPL3-mediated shedding are in principle catalytically active, outside the cell they lack nucleotide- or lipid-linked sugar donor substrates that seem to exclusively occur intracellularly [80, 81]. Consequently, SPPL3 acts as a negative regulator of cellular protein glycosylation and increased SPPL3 expression results in hypoglycosylated secreted and membrane proteins, while reduced SPPL3 expression leads to hyperglycosylation [48]. Consequently, SPPL3 is a potent switch to regulate protein glycosylation and thus, also the composition of the extracellular matrix. Changes in the glycan composition of membrane proteins affect physiological processes like signalling and cell growth, but also pathological processes like angiogenesis, tumour metastasis and autoimmunity [82–84]. Based on the fact that proteolytic cleavages are irreversible, it must be assumed that SPPL3 expression under physiological conditions is tightly regulated. Interestingly, also SPPL2c has an impact on protein glycosylation upon overexpression [31]. However, this is based on the retention of glycosyltransferases in the ER following cleavage of SNARE proteins. Therefore, in contrast to SPPL3, SPPL2c overexpression predominantly results in accumulation of immature glycoproteins rather than in changed glycan patterns [31]. In vivo, sperm from SPPL2c-deficient mice exhibit altered glycan patterns [18].

## Pathophysiological functions of SPP/SPPL proteases

All these examples illustrate that SPP/SPPL proteases have an impact on several central cellular pathways by controlling the level of substrate proteins in cellular membranes. This qualifies them as regulatory switches and may have major implications in pathophysiological conditions and diseases. However, so far the regulation of these proteases is not understood. It is highly conceivable, though not documented yet, that the activity of SPP/SPPL proteases is actively modulated in certain cellular states and/or in response to certain signals. In addition, when considering protease functions in an in vivo context, the tissue expression patterns of the respective protease as well as of the potential substrates are critical determinants. Only when both are co-expressed in a certain cell type cleavage can occur. The distribution of the two closely related family members SPPL2a and SPPL2b showed substantial differences when analysed by Western blotting in major murine tissues [64]. SPPL2a was detected in all tissues analysed, however, with the lowest abundance in brain. In contrast, SPPL2b showed a much more focused expression pattern significantly dominated by brain and lower abundance in the lymphatic system. In liver, kidney, lung and intestine SPPL2b was not reliably detected with the available antibody, indicating, if at all, low expression of this protease. Presence of SPPL3 at the protein level has been demonstrated in murine brain, lung, spleen and embryonic fibroblasts [48]. When analysed in sorted splenocytes, SPPL3 levels were much higher in NK cells than in B cells or T cells [85]. In general, a rather ubiquitous expression of SPPL3 is also supported by previous transcriptional data from human tissues [15]. The same holds true for SPP [15], which has not yet been directly compared at the protein level in different tissues. However, its documented endogenous presence in several standard and carcinoma cell lines including HEK293, HCT116, A549 and DU145 [18, 50, 51] as well as in murine testis [18] strongly suggest that SPP is a general constituent of human and murine cells.

**Fig. 4:**
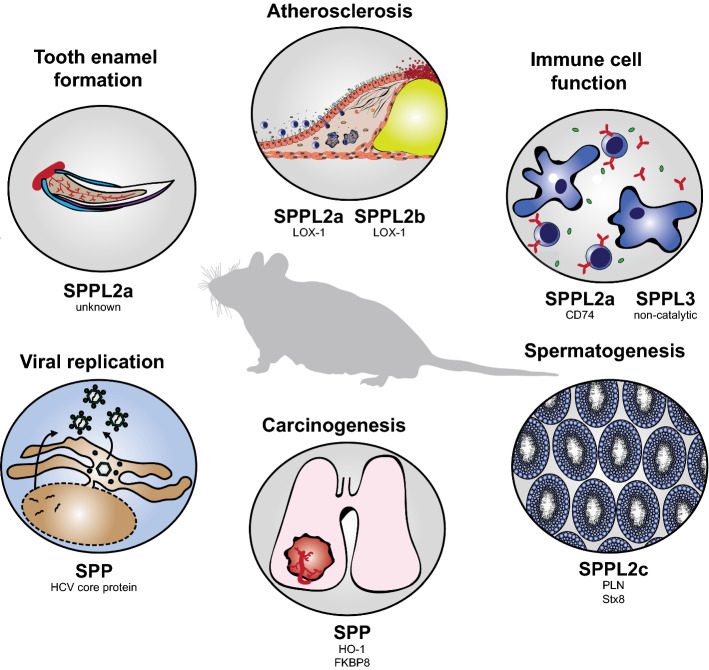
Pathophysiological functions of SPP/SPPL proteases. Summary of the proteases’ in vivo functions based on phenotypes of the respective protease-deficient mouse models. For each pathophysiological process, the implicated protease and the responsible substrate (if known) are listed

Until recently, SPPL2c has remained the most neglected member of the SPP/SPPL family. Even though initial reports suggested that human SPPL2c might be an ER-resident protein, SPPL2c was considered a potential pseudogene due to its intronless gene structure [15] without any proof of endogenous expression or catalytic activity. Two recent publications clarified the status of SPPL2c as catalytically active enzyme [18, 31]. In contrast to the other rather broadly expressed family members, expression of SPPL2c is restricted to the testis both on mRNA and protein level in man and mice [18] arguing for a specific function in this tissue context. Our current state of knowledge about in vivo function of the individual SPP/SPPL proteases and—as far as this is known—the associated substrates is summarised in Fig. 4. This largely results from the analysis of SPP/SPPL-deficient mouse models and only in the case of SPPL2a from the characterization of human patients [68].

### Immune system

SPPL2a and SPPL3 play a critical role for the differentiation and homeostasis of immune cells—however of different cell types. Whereas SPPL2a is required for differentiation of B lymphocytes and certain subsets of dendritic cells [57, 65, 66, 68], SPPL3 deficiency impairs NK cell maturation and function [85]. SPPL2a-deficient or mutant mice display a defect of splenic B cell maturation characterised by an arrest at the transitional stage 1. Consequently, mature B cells are depleted and humoral immune responses are significantly impaired [57, 65, 66]. Furthermore, also the abundance of conventional dendritic cells (cDCs) is reduced, whereas plasmacytoid dendritic cells (pDCs) are not affected [57, 65, 66]. These phenotypes are significantly alleviated in SPPL2a-CD74 double-deficient mice clearly identifying CD74 NTFs, which accumulate in the absence of SPPL2a, as the underlying mechanism [57, 65]. Immortalized human B cells with genetic SPPL2a deficiency also accumulate CD74 NTFs indicating that the requirement of SPPL2a for the turnover of this fragment is conserved in humans [67]. However, the potential consequences of B and dendritic cell differentiation and function in humans remained elusive until recently when Jean-Laurent Casanova and co-workers reported on three patients from two different families harbouring splice site mutations in the *SPPL2a* gene, which abolished expression of this protease [68]. CD74 NTF accumulation was evident in B cells and monocytes in the blood of these individuals. Clinically, the SPPL2a-deficient patients suffer from Mendelian Susceptibility to Mycobacterial Disease (MSMD). This describes a genetic predisposition to develop invasive infections from low-virulent mycobacteria like the tuberculosis (TB) vaccination strain Bacille Calmette-Guérin (BCG) which are usually well-controlled by immuno-competent individuals [86]. Immuno-phenotyping revealed significantly reduced frequencies of CD1c^+^CD11c^hi^HLA-DR^hi^ cDC2, but only marginally reduced frequencies of CD141^+^Clec9a^+^ cDC1 and normal pDCs [68]. This demonstrates a subset-selective loss of cDCs. Since early DC precursors were reduced as well, SPPL2a-deficiency seems to already affect pre-DCs, when expression of MHCII and CD74 is initiated [68]. Though T cell development and activation were not generally affected, the patients exhibit a defect in mycobacterium-specific IFN-γ production by memory CD4^+^ T cells. This may reflect that the depleted cDC2 cells in humans are potent producers of the cytokine IL-12 which is critical for priming of T cells and induction of a T_H_1 polarised T cell response. Furthermore, an important role of this cell population in antimycobacterial immunity has been suggested previously by MSMD cases with other genetic etiologies so that cDC2 may represent the key cells for presentation of mycobacterial antigens to CD4^+^ T cells [68]. The subset-selective depletion of dendritic cells in the human patients correlates well with the phenotype of *SPPL2a*^*−/−*^ mice, which also showed an enhanced susceptibility to mycobacterial infections in different models. Upon infection with BCG mycobacteria, they developed greater splenomegaly and exhibited higher BCG colony-forming unit (CFU) counts [68]. Following aerosol infection with high-virulent *M. tuberculosis* the SPPL2a-deficient mice succumbed more rapidly and exhibited higher pathogen burden in the lungs [68]. Interestingly, they exhibit increased pulmonary inflammation and neutrophil influx, which, however, does not convey protective immunity. Altogether, this indicates that *SPPL2a*^*−/−*^ mice represent a suitable disease model for human SPPL2a deficiency. In this context it was a surprising finding that the three patients did not exhibit a relevant impairment of B cell differentiation [68]. Frequencies of CD19^+^ B cells including transitional, naïve and memory B cells were normal. However, total plasma IgG was slightly reduced in all three patients, whereas total IgM was increased in two of the individuals. This is in contrast to SPPL2a-deficient mice where functionally mature B cells as well as immunoglobulins of all subclasses were massively reduced [68]. This striking phenotypic discrepancy with regard to B cells is currently unexplained. It is well established that in human genetic diseases genotype–phenotype correlations can vary. Therefore, it will be important to see if also in other cases potentially identified in the future human SPPL2a-deficiency does not affect B cells. Upon treatment of different human immune cell subsets with an SPPL2a inhibitor, cDC2 exhibited the highest CD74 NTF accumulation exceeding that of B cells by factor 2 [68]. This could indicate that in human B cells the turnover of CD74 and thus their potential to accumulate NTFs is lower than in DCs so that a critical threshold is not reached. It could also be that human B cells are less susceptible to the effects triggered by the CD74 fragment than murine B cells. Furthermore, in humans two additional CD74 isoforms with an extended N-terminus are present [47] and may contribute to the phenotypic differences between SPPL2a-deficient mice and humans. Despite the evidence that the CD74 NTF causes the immunological phenotypes associated with SPPL2a-deficiency, the molecular cascade triggered by this fragment still remains to be determined. The work in murine B cells has revealed a capability to impair membrane trafficking and signal transduction. How these findings may be transferred to the processes in dendritic cells remains to be analysed.

Since constitutive SPPL3-deficient mice exhibit increased lethality [48, 85], SPPL3 function in the hematopoietic system has been examined in conditional *SPPL3* knockout mice bred with *Vav1-iCre* and *NKp46-iCre* alleles [85]. Both strains exhibited an impaired proliferation of CD27^+^CD11b^−^ NK cell precursors in the bone marrow and reduced survival of CD27^+^CD11b^+^ and CD27^−^CD11b^+^ NK cells. As a result, peripheral NK cells are significantly depleted in the spleen and liver of these mice. The remaining NK cells show reduced levels of different surface receptors as well as reduced cytotoxic activity. Based on the specificity of the *NKp46-iCre* allele, these findings demonstrate a cell autonomous role of SPPL3 in NK cells [85]. This depends on the proteolytic activity since an inactive SPPL3 D271A allele was unable to rescue the phenotype. So far, the underlying substrate is unknown. *SPPL3*^*−/−*^ NK cells accumulate the glycosyltransfersase MGAT5 as it has been described for other cell types, where this leads to a hyperglycosylation phenotype [48]. However, lectin binding experiments rather demonstrated a reduction of complex glycosylation at the plasma membrane of *SPPL3*^*−/−*^ NK cells. This may indicate that additional mechanisms and also NK cell-specific substrates are involved [85]. This is supported by *Vav1-iCre SPPL3 Ko* mice with a more generalised depletion of SPPL3 in the hematopoietic system. There, numbers of B cells and T cells are not affected, although the protease is expressed in the respective cells in wild type mice [85].

### Atherosclerosis

Recently, an important impact of SPPL2a and SPPL2b on the development of atherosclerosis was uncovered [39]. Atherosclerosis was induced in *wild type* and *SPPL2a/b*^*−/−*^ mice via overexpression of a gain-of-function mutant of the Proprotein Convertase Subtilisin/Kexin type 9 (PCSK9) in order to deplete the LDL receptor in combination with a high fat/high cholesterol diet. By this means, hypercholesterolemia and atherosclerotic plaque development are induced to a similar degree as in the widely employed *Ldlr*^*−/−*^ model [87]. In order to avoid confounding effects related to the immunodeficiency associated with SPPL2a deficiency, all mice were reconstituted with *wild type* bone marrow prior to the experiment [39]. Atherosclerosis was significantly increased in *SPPL2a/b*^*−/−*^ mice as compared to controls based on the development of larger and more advanced plaques. Surprisingly, the induced hypercholesterolemia of SPPL2a/b double-deficient mice was ~ 30% lower than in *wild type* mice. Since circulating cholesterol is a major risk factor for plaque development, it may be speculated that this experiment has rather underestimated the pro-atherogenic phenotype of *SPPL2a/b*^*−/−*^ mice and that the difference in plaque burden may have been even more pronounced if plasma cholesterol levels had been equivalent in both genotypes. In addition to the increased size, also collagen deposition was increased in plaques of SPPL2a/b-deficient mice. At the molecular level, activation of the ERK and p38 MAP kinase pathways and expression of the adhesion molecule ICAM-1 were enhanced in these mice [39].

Altogether these results demonstrate that SPPL2a/b confine and limit the development of atherosclerosis, which leads to cardiovascular diseases and therefore represents a major cause of morbidity and mortality [88]. This athero-protective role of SPPL2a/b could be correlated to the processing of the LOX-1 NTF for which both proteases have a synergistic function in vivo [39]. LOX-1 plays a pivotal role in the onset and development of atherosclerosis, mediated by the production of reactive oxygen species, pro-inflammatory cytokines and the expression of cell adhesion molecules such as ICAM-1 and VCAM-1. It is expressed mostly in endothelial cells, but can also be found in macrophages, smooth muscle cells and platelets [70, 89]. Furthermore, an increased development of atherosclerosis is observed in mice overexpressing LOX-1 [90]. Aortae from *SPPL2a/b*^*−/−*^ mice exhibit a significant accumulation of LOX-1 NTFs. As discussed above, this fragment can influence cellular signal transduction by different mechanisms. In endothelial cells, overexpression of the LOX-1 NTF led to the activation of MAP kinases and induced expression of several pro-atherosclerotic genes, including ICAM-1 [39]. This correlates strongly with the observations from atherosclerotic lesions of SPPL2a/b-deficient mice. Thus, the phenotype of *SPPL2a/b*^*−/−*^ mice was consistent with higher LOX-1 activity, likely driven by the accumulation of the LOX-1 NTF. Of note, the LOX-1 NTF is present at detectable levels in the aorta of wild type mice and its abundance increases in *Ldlr*^*−/−*^ mice subjected to an atherogenic diet [39]. SPPL2a/b mediate the turnover of this fragment, terminate signaling and by this means control the development of atherosclerosis. Since the human LOX-1 NTF can be processed by SPPL2a/b and the proteases are present in human atherosclerotic lesions [39], these mechanisms are most likely conserved in humans.

### Germ cell differentiation

To date, only the function of SPPL2c, which shows a testis-specific expression, has been analysed in the context of germ cell biology [18, 31]. However, also SPP, SPPL2a and SPPL2b are expressed in murine testis at the protein level ([18] and unpublished observations) and can be anticipated to have a function in developing male germ cells. The distribution of SPPL2c in the testis is not uniform. It is expressed in spermatids and can be most prominently found in elongated spermatids which represent a late progenitor stage of mature spermatozoa [18]. In these cells, two different variants of SPPL2c can be detected as mono-glycosylated glycoproteins in membranes of the ER [18]. Both variants differ only in the amino acid composition of their cytosolic C-terminus that is truncated in the smaller one originating from differential splicing. *SPPL2c*^−/−^ mice show a decreased testis/body weight ratio, which is associated with a reduction of elongated spermatids. Morphology of mature epididymal spermatozoa was not different from wild type mice. However, the motility of SPPL2c-deficient sperm was significantly reduced [18]. This was a surprising finding, since also in wild type mice SPPL2c is not present in mature spermatozoa reflecting that after completion of meiosis spermatids have to undergo a major reorganisation including the loss of cytoplasmic and ER structures in order to become mature sperm. This indicates that the function of SPPL2c in spermatid lays a foundation for optimal motility and fitness at a differentiation stage after the protease itself has already been eliminated from the cell.

Despite this impact on sperm differentiation and function, *SPPL2c*^−/−^ mice only show a mild reproductive phenotype [18]. When paired with wild type females, SPPL2c-deficient males produce litters of comparable size to wild type controls. In contrast, litter sizes of homozygous *SPPL2c*^*−/−*^ X *SPPL2c*^*−/−*^ breedings were significantly reduced indicating a subfertility that only manifests when both partners are SPPL2c-deficient. This argues for an additional role of SPPL2c in the female reproductive tract where the protein so far could not be detected [18]. However, it cannot be excluded that also here expression of SPPL2c is restricted to a certain cell type or even a specific maturation stage, e.g. during oogenesis, which might limit detection of this protein in the respective organs.

In order to understand the molecular basis of the phenotypes induced by SPPL2c deficiency, insights into the substrate spectrum of SPPL2c were obtained by mass spectrometry-based proteomic analysis of either SPPL2c overexpressing HEK cells [31] or total membrane preparations of wild type or SPPL2c-deficient testis [18]. While overexpression of SPPL2c led to reduction in levels of a total of 25 type II and TA proteins [31], under physiological conditions only the SNARE protein Syntaxin-8 (Stx8) and the Ca^2+^-regulator phospholamban (PLN) were significantly accumulated upon SPPL2c-deficiency indicating a potential protease-substrate relationship (Fig. 3) [18]. However, it cannot be excluded that the highly restricted expression of this protease in elongated spermatids prevented identification of additional SPPL2c substrates, because effects were “diluted” in the preparations from total testis. Nevertheless, the identified substrates strongly correlate with the phenotype of SPPL2c overexpressing HEK cells as well as *SPPL2c*^−/−^ mice.

Overexpression of SPPL2c in HEK cells caused a disintegration of Golgi compartments connected with a hypoglycosylation of various glycoproteins caused by the retention of glycosyltransferases in the ER [31]. In agreement with these changes in cell-based experiments, SPPL2c-deficient mice show an altered glycan finger-print in mature spermatozoa as well as a less compact organisation of the Golgi apparatus in spermatids as judged from immunohistochemical staining of the Golgi protein Cab45 [31]. Therefore, it seems likely that SPPL2c participates in the remodelling of maturing spermatids. In addition to the removal of organelles and cytoplasm, this includes the formation of the acrosome which at least partially originates from Golgi compartments [91].

In addition to this cell structure-regulating function, the loss of SPPL2c also causes a reduction of cytoplasmic Ca^2+^ levels specifically in elongated spermatids which might be explained by increased levels of PLN under these conditions [18]. However, especially due to contradicting reports about expression and function of SERCA isoforms in the testis [92, 93] the precise role of PLN in this organ remains enigmatic. Nevertheless, alterations in Ca^2+^ handling and reduced Ca^2+^ levels in SPPL2c-deficient spermatids might be linked to the reduced motility of mature epididymal spermatozoa from *Sppl2c*^−/−^ mice [18], since sperm movement is considered to be Ca^2+^-dependent [94].

### Cancer

With their potential to influence cellular signalling, it may be hypothesised that SPP/SPPL proteases also have an impact on neoplastic cells. This has so far only been investigated for SPP. SPP is strongly upregulated in several types of cancer including breast, colon and lung tumours [95] as well as glioblastoma multiforme (GBM) [96]. In many cases, expression of SPP correlates with the progression and malignancy of the tumour [95, 96] making it a potential biomarker for staging of these diseases. While in some cases the pro-tumorigenic function of SPP could be delineated to processing of individual substrates like HO-1 [51] or FKBP8 [95], the molecular mechanism underlying the pro-tumorigenic function of SPP in EGFRvIII glioblastoma is not as well understood. This type of GBM expresses a constitutively active truncated mutant of the epithelial growth factor receptor (EGFR) responsible for uncontrolled cell growth of glia cells [97]. In addition, EGFRvIII GBM cells secrete increased amounts of different cytokines including TGF-β1 and TGFα but also other hormones like Insulin which promote tumour growth in a paracrine way [96]. Knockdown of SPP significantly reduced the upregulated cytokine production in EGFRvIII U87 glioblastoma cells. Importantly, reduced TGF-β1 secretion by EGFRvIII U87 cells upon knockdown of SPP resulted in less pro-tumorigenic SMAD signalling in normal glioblastoma cells treated with the supernatant of these cells thereby reducing their invasive capacity [96]. This pro-tumorigenic effect of SPP could be recapitulated in an orthotopic tumour growth model, where control- or SPP-siRNA transduced tumour cells were injected into the brain of wild type Balb/c nude mice. Under this experimental regime, knockdown of SPP significantly prolonged the survival of these animals and reduced tumour volumes [96]. The prolonged survival correlated with reduced secretion of TGF-β1 and TGFα as well as decreased phosphorylation of SMAD2 in the brain suggesting reduced cytokine secretion by the tumour cells upon reduced SPP expression. However, the precise function of SPP in this process has not been clarified. Since it was shown that SPP is capable of cleaving SNARE proteins [52], it is tempting to speculate that altered trafficking in the early secretory pathway might be involved.

While the substrates responsible for the pro-tumorigenic effect of SPP in GBM currently remain elusive, a crucial involvement of the newly identified SPP substrate FKBP8 was demonstrated in breast and lung cancer cell lines [95]. Here, knockdown of SPP reduces growth, migration and invasion in vitro. A SILAC-based proteomic analysis of a lung cancer cell line treated with siRNA against SPP identified FKBP8 as a novel SPP substrate. FKBP8 is an ER-resident tail-anchored protein, which depends on the intramembrane cleavage by SPP for its degradation by the proteasome [95]. Therefore, in the absence of SPP, FKBP8 turnover is impaired and its cellular levels increase. FKBP8 can function as an inhibitor of the mTOR pathway, which was suppressed in SPP-depleted cells [95]. This could be reversed by additional knockdown of FKBP8 providing a link between this substrate and the lower mTOR signalling upon SPP knockdown. Importantly, these findings were recapitulated in vivo using a xenograft model. Stable knockdown of SPP in MDA-MB-231 breast cancer cells attenuated tumour growth after injection into mice. However, an additional knockout of FKBP8 abolished this effect and tumours grew similar to those from control cells [95].

In addition to FKBP8, SPP-mediated cleavage of the TA protein HO-1 has also been linked with cancer progression [51]. However, in this case the pro-tumorigenic effect of SPP is based on the release of a biologically active cleavage fragment into the cytosol, which is a prerequisite for its translocation into the nucleus. HO-1 nuclear localisation has been detected in several types of cancer and has been linked with tumour progression. SPP knockdown or inhibition reduces nuclear localisation of HO-1. In HeLa and H1299, overexpression of wild type HO-1 and a truncated form without the C-terminal transmembrane segment enhanced cell proliferation and migration suggesting that this was dependent on the nuclear form of HO-1. Corresponding effects on tumour size and number of metastases were observed following transplantation of the cell lines expressing the cytosolic/nuclear form of HO-1 into nude mice [51]. The catalytic activity of HO-1 seems to be dispensable for this pro-tumorigenic action. However, the molecular function of HO-1 in the nucleus is currently unresolved.

Beyond SPP, very little is known about a potential function of SPPL proteases in cancer. SPPL2a has been implicated in the processing of Teneurin-1/ODZ1 [59]. ODZ1 is a large type II transmembrane protein with physiological functions especially in the developing brain. Its expression in GBM tumours correlates with their aggressiveness and reduced survival. In a wound-healing assay in vitro, ODZ1 enhances the migration and invasive capacity of GBM cells. This effect depends on the nuclear translocation of the ODZ1 ICD, which is able to activate the RhoA-ROCK pathway by triggering a complex transcriptional program. It was shown that knockdown of SPPL2a in GBM cells reduces nuclear translocation of the ODZ1 ICD [59] suggesting a direct role of this protease in cleaving ODZ1. If this is the case, inhibition or knockdown of SPPL2a should be able to attenuate the pro-invasive downstream mechanisms of ODZ-1 expression. However, this has not been further explored yet.

### Viral infections

As recently reviewed [13], SPP plays a role in different viral infections by being involved in the processing and thereby maturation of different viral proteins. By this means, the SPP activity of the host cell is a critical factor for viral replication. The most intensively investigated example is the Hepatitis C virus (HCV) core protein which is required for subsequent assembly of viral particles at lipid droplets and therefore finally also virus production and propagation [98–101]. Loss of SPP leads to an impaired maturation of the HCV core protein and the subsequent TRC8-dependent degradation of the immature precursor through the ERAD pathway [102, 103]. Due to its essential role in the viral life cycle, pharmacological inhibition of SPP can suppress HCV replication in cultured cells [104]. Importantly, independent of virus replication expression of the HCV core protein is at least in part responsible for the HCV-associated liver pathology, as demonstrated by HCV core transgenic mice [102, 104]. SPP haploinsufficiency or treatment with SPP inhibitors significantly ameliorated the degree of liver steatosis and the development of insulin resistance in these mice [102, 104]. This is based on the proteasomal degradation of the HCV core protein when its maturation is blocked due to lack of cellular SPP activity.

Also the Bunyamwera orthobunyavirus glycoprotein precursor is processed by SPP in cooperation with signal peptidase [105]. The replication of *Bunyamwera virus* in cells with sh-RNA-silenced SPP expression was about tenfold lower than in control cells. Propagation of other viruses from the same family was assessed in the same experimental set-up. A significant inhibitory effect was observed on *Orthobunyavirus genus* replication, which was much less pronounced for *Phlebovirus genus*. It will be an interesting question if similar effects can also be achieved with pharmacological SPP inhibition.

Furthermore, SPP also supports replication of the Herpes simplex-1 (HSV-1) virus, a common human pathogen. SPP was shown to bind to Glycoprotein K (gk), a virion envelope protein of HSV-1 [106], although it is not clear if this involves any proteolytic cleavage. SPP knockdown blocks HSV-1 replication in vitro [106]. A similar effect was achieved by expressing catalytically inactive SPP mutants [106] or by treatment with SPP inhibitors like (Z-LL)_2_-ketone [107]. This compound also reduced HSV-1 replication in vivo when topically applied before and during ocular infection of mice. Recently, the role of SPP for HSV-1 infectivity in vivo was further confirmed in a genetic model [108]. Since constitutive SPP knockout mice are lethal [102], a floxed SPP locus in combination with a tamoxifen-inducible Cre recombinase was employed [108]. Mice with induced SPP deletion exhibited significantly less HSV-1 replication in their eyes. Furthermore, also the infiltration of immune cells as well as the latency of the virus was significantly reduced [108].

### Further functions

An additional phenotype of SPPL2a-deficient mice not falling in any of the categories above is a defect in tooth enamel generation [109]. *SPPL2a*^*−/−*^ mice exhibit chalky white teeth with an incompletely mineralised tooth enamel resulting in insufficient mechanical strength of this material. The enamel-producing ameloblasts, in particular during the later stages of enamel production, show major morphological alterations and degenerate. The function of SPPL2a in ameloblasts and any potential substrates of SPPL2a causing this phenotype currently remain enigmatic.

In general, it should be noted that the above synopsis of pathophysiological functions of SPP/SPPL intramembrane proteases most likely is incomplete considering that constitutive SPPL3 and SPP knockout mice show enhanced or complete lethality impeding their analysis and that comprehensive characterisation of conditional models is yet pending.

## Recent advances in SPP/SPPL inhibitor development

Based on the growing interest in inhibition of SPP/SPPL protease family members especially for the modulation of the adaptive immune system but also for treatment of viral and plasmodium infections, a substantial effort was invested in the generation of small molecule inhibitors for these proteases [110–112]. This process significantly profited from existing compounds initially generated for inhibition of the related γ-secretase for treatment of Alzheimer’s disease and leukaemia [113]. While some of these inhibitors appear to selectively target γ-secretase and spare the SPP/SPPL family, others like L-685,458 and LY-411575 also inhibit processing of SPP/SPPL substrates even though with different efficiency for individual proteases [13, 114, 115]. In this context, it was an interesting observation that SPPL2c activity is targeted by DAPT [18], a well-established γ-secretase inhibitor (GSI) that spares all other SPP/SPPL proteases [13] strongly arguing for major differences between SPPL2c and the other family members.

In fact, some GSIs have already been successfully applied to target SPP in animal models of protozoal/viral infections. Inhibition of Plasmodium falciparum SPP leads to a marked reduction of parasite growth [116]. Treatment of *Plasmodium falciparum* or *Toxoplasma gondii* with 10 µM of the dibenzoazepine GSIs Ly-411575 and YO-01027 results in significant killing of the protozoa in cell culture models [104]. In line with these findings, a single dose of YO-01207 applied 24 h post-infection prolongs survival of *Toxoplasma gondii*-infected mice from 5 to 6 days, indicating that indeed inhibition of parasite SPP might represent a therapeutic approach in vivo [104]. Similarly, YO-01027 was able to prevent HCV replication in cultured liver cells as well as to ameliorate the liver pathology in mice overexpressing the HCV core protein, targeting in this case the SPP activity of the host cell [104]. Importantly, in cell culture models the antiviral effect of YO-01207 and LY-411575 was seen for all known genotypes of HCV and was much less susceptible to the development of drug-resistance than other antivirals, highlighting SPP-targeting small molecules as promising anti-HCV drug candidates [104]. Some GSIs including BMS-906024 and RO4929097 also potently inhibit SPPL2a and induce CD74 NTF accumulation upon application to B cells [114].

Despite the clear potential of GSIs for therapeutic inhibition of SPP and possibly also SPPL2a, these drugs are certainly not ideal for treatment of patients, since they target several SPP/SPPL proteases and, most importantly, also γ-secretase. GSIs cause significant adverse effects like induction of skin cancers and gastrointestinal side-effects making them fail in clinical trials for treatment of Alzheimer’s disease [117, 118]. Therefore, the availability of γ-secretase sparing SPP/SPPL protease inhibitors would be essential. For a long time, the only SPP/SPPL inhibitor not targeting γ-secretase is (Z-LL)_2_-ketone [13], which inhibits SPP, SPPL2a and SPPL2b, but has little or no impact on SPPL3 [30] and SPPL2c [18]. In addition, (Z-LL)_2_-ketone has a low effectivity and unfavourable physicochemical properties. Furthermore, though sparing γ-secretase its specificity is limited as it can also inhibit the cysteine proteases cathepsin B and L at higher concentrations [119]. Therefore, the development of novel SPP/SPPL family and ideally, individual protease-specific inhibitors represents an absolute prerequisite for potential therapeutic use. Using several independent strategies, Novartis has generated small molecule inhibitors specifically targeting SPPL2a, which might represent an interesting target for treatment of autoimmune diseases based on its role in dendritic cells and also B cells, though the impact on the latter is not confirmed in humans [110–112]. For high throughput compatible assessment of compounds, these studies employed an high-content imaging assay to monitor activity of SPPL2a [110]. This was based on a reporter cell line with stable expression of SPPL2a in combination with inducible expression of a GFP-tagged reporter construct [110]. This comprised a membrane-bound fragment of TNFα, which can be directly cleaved by SPPL2a without the need of preceding ectodomain processing and was fused to GFP at its N-terminus via a nuclear localisation signal (NLS). Cleavage of this construct by the co-expressed protease results in constitutive nuclear transport of the GFP-containing cleavage fragment, whereas inhibition of the protease leads to a retention of the substrate molecule in the plasma membrane and prevents its accumulation in the nucleus [110]. This assay was employed in a multi-parametric image-based screen of a library containing 1.4 million compounds. For two hits of the screen, the authors confirmed inhibition of endogenous SPPL2a in a murine B cell line based on CD74 NTF accumulation. As determined in respective cell based assays, the effect of these candidates on γ-secretase and also other members of the SPP/SPPL family like SPP and SPPL2b was much lower than on SPPL2a indicating their potential as scaffolds for the development of SPPL2a-specific inhibitors [110]. Optimisation of an additional hit compound from this screen resulted in the generation and characterisation of the orally active hydroxyethylamine-based SPPL2a inhibitor SPL-410 as recently reported by Velcicky et al. [112]. By variation of the different substituents, the IC_50_ in a cell-based SPPL2a assay could be lowered from 4.3 to 0.009 µM of the optimised compound SPL-410. IC_50_ values against γ-secretase, SPP and SPPL2b were around 1.3 µM, 0.65 µM and 0.27 µM, respectively, indicating a certain selectivity of this compound for SPPL2a [112]. However, the activity of SPL-410 against SPPL2c and SPPL3 has not been reported yet. In general, oral application of a single dose (10 mg/kg body weight) of SPL-410 to mice was able to induce the accumulation of the CD74 NTF in the spleen. However, a rather high plasma protein binding limits the potency and applicability of this inhibitor in vivo making further rounds of optimisations necessary [112].

In an alternative approach, derivatisation of the γ-secretase inhibitor LY-411575, which also inhibits SPPL2a, was performed [111]. Optimisation of the different substituents was aimed at increasing selectivity of the SPPL2a inhibition over that of γ-secretase and to improve the pharmacokinetic profile. The optimised compound SPL-707 showed a 79-fold and 23-fold selectivity over γ-secretase and SPP, respectively. In contrast, the IC50 for inhibition of SPPL2b was only 2.7-fold higher than that for SPPL2a [111]. Inhibition of SPPL2a by SPL-707 is reversible as the CD74 NTF accumulation was completely eliminated in a washout experiment in B cells 2 h after withdrawal of the inhibitor. As described above for SPL-410, also SPL-707 was capable of inducing CD74 NTF accumulation in mice following a single oral dose. More importantly, when administered over 11 days this compound also induced changes in immune cell populations reminiscent of the phenotype of SPPL2a-deficient mice [111]. These were characterised by a reduction of total and mature B cells as well as cDCs. As expected from its specificity profile, SPL-707 did not lead to a detectable impairment of Notch signalling in treated mice. Importantly, this study demonstrated for the first time that the immunological phenotype of *SPPL2a*^*−/−*^ mice does not require absence of the protease during development, but instead can be induced with an inhibitor in adult mice, which is a prerequisite for therapeutic SPPL2a inhibition. How successful this concept will be in humans in order to treat autoimmunity will require careful evaluation. Altogether, these studies have demonstrated that the development of γ-secretase-sparing SPP/SPPL inhibitors even with a certain selectivity for individual family members can be achieved [110–112].

## Conclusions

Since the identification of SPP in 2002 and the subsequent discovery of the SPPL proteases, our understanding of this protease family has greatly increased. Initially, intramembrane proteases were considered as an unspecific proteasome of the membrane. Though we still do not understand which determinants are recognised by SPP/SPPL proteases and define a substrate, it has become clear that cleavage by these proteases is highly selective. Beyond the direct protease–substrate interaction, the restricted tissue expression patterns of some substrates and proteases as well as their subcellular localisations add an additional level of specificity in vivo. Several examples have been identified where SPP/SPPL proteases, based on specific cleavage events, act as regulatory switches of signal transduction, membrane trafficking and protein glycosylation with important functional consequences in a cellular context but also in vivo. Since the different cleavage events can have such a critical impact, it has to be assumed that they take place in a tightly controlled way. So far, it is entirely unclear how this is achieved and it will be one of the key questions for further research to unravel regulatory mechanisms of the proteases themselves. With regard to their involvement in pathophysiological processes, SPP/SPPL proteases play an important role in immune defence. However, their recently discovered involvement in atherosclerotic plaque development and germ cell biology has significantly widened the spectrum. It can be anticipated that this will be further expanded when the in vivo functions of SPP and SPPL3, where constitutive knockout mice are lethal, are dissected using conditional mouse models. The recent identification of SPPL2a-deficient patients has certainly confirmed the pathophysiological relevance of this protease. However, the reason for the phenotypic differences observed between mice and men requires clarification. Beyond being of interest from a mechanistic point of view, this question is important also from a translational perspective. Recent efforts in inhibitor development have indicated that it is possible to develop inhibitors of SPPL2 proteases sparing γ-secretase and SPP. However, to better judge their therapeutic potential and the spectrum of indications a precise definition of the in vivo function of SPPL2a in humans will be critical. To what extent the other SPP/SPPL members may also have potential as therapeutic targets further work will show.
